# The retinoid X receptor from mud crab: new insights into its roles in ovarian development and related signaling pathway

**DOI:** 10.1038/srep23654

**Published:** 2016-03-24

**Authors:** Jie Gong, Chencui Huang, Ling Shu, Chenchang Bao, Huiyang Huang, Haihui Ye, Chaoshu Zeng, Shaojing Li

**Affiliations:** 1 College of Ocean and Earth Sciences, Xiamen University, Xiamen 361102, China; 2 School of Life Sciences, Nantong University, Nantong 226007, China; 3 Fujian Collaborative Innovation Center for Exploitation and Utilization of Marine Biological Resources, Xiamen 361102, China; 4 College of Marine and Environmental Sciences, James Cook University, Townsville, Queensland 4811, Australia

## Abstract

In arthropods, retinoid X receptor (RXR) is a highly conserved nuclear hormone receptor. By forming a heterodimeric complex with the ecdysone receptor (EcR), RXR is known to be vital importance for various physiological processes. However, in comparison to EcR, the RXR signaling pathway and its roles in crustacean reproduction are poorly understood. In the present study, the RXR mRNA was detected in the ovarian follicular cells of mud crab *Scylla paramamosain* (SpRXR) and during ovarian maturation, its expression level was found to increase significantly. *In vitro* experiment showed that both SpRXR and vitellogenin (SpVg) mRNA in the ovarian explants were significantly induced by 20-hydroxyecdysone (20E) but not methyl farnesoate (MF). However, differing from the *in vitro* experiment, injection of MF in *in vivo* experiment significantly stimulated the expressions of SpRXR and SpVg in female crabs at early vitellogenic stage, but the ecdysone and insect juvenile hormone (JH) signaling pathway genes were not induced. The results together suggest that both MF and SpRXR play significant roles in regulating the expression of SpVg and ovarian development of *S. paramamosain* through their own specific signaling pathway rather than sharing with the ecdysone or the insect JH.

In arthropods, many important physiological processes, including molting, appendage regeneration and reproduction, are regulated by the biological active forms of ecdysone (e.g. 20-hydroxyecdysone, 20E), a group of steroid hormones^1^. To mediate these processes, ecdysone needs to bind to the ecdysone receptor (EcR) first to activate the early response genes^2^. EcR is known belonging to the superfamily of nuclear receptor and can form a heterodimer complex with another nuclear receptor, the retinoid-X receptor (RXR), which is also known as ultraspiracle (USP) in insects^3^,^4^,^5^. Of all nuclear receptors, RXR is the most widely found one and reportedly targeting multiple signaling pathways^2^,^6^. Other than with EcR, RXR also serves as the partner in other heterodimeric receptor complexes, including the thyroid hormone receptor and the retinoic acid receptor^7^. Therefore, RXR plays essential roles in various physiological processes of arthropods. For example, RXR has reportedly been involved in the regulation of vitellogenin and ovarian development^6^,^8^,^9^,^10^,^11^,^12^,^13^,^14^, cuticle production and molting^4^,^6^,^9^,^15^, embryonic development^16^,^17^ and limb regeneration^2^,^18^,^19^ in various arthropods.

RXR can be activated by various ligands. In vertebrates, RXR can bind to 9-cis retinoic acid (9-cis RA) and tributyltin, both considered the nature ligands of RXR, with high affinity^20^,^21^. However, USP from insects as the homolog of RXR is reportedly not sensitive to known RXR ligands, such as 9-cis RA or other RA metabolites, suggesting that it is functionally distinct to vertebrate RXR^22^,^23^. In fact there is no hormone ligand that has been conclusively established for USP, although JH might directly modulate the activity of the EcR⁄USP complex in insects^24^. Different from vertebrates and insects, in crustaceans JH has not yet been conclusively identified, and the exogenous 9-cis RA and JH also could not activate the RXR or EcR⁄USP (RXR) complex^25^,^26^,^27^. While it is still contentious regarding what are the ligands of RXR in crustaceans, the methyl farnesoate (MF), a unepoxidated form of the insect JH, has be suggested as a candidate ligand for RXR of crustaceans^2^,^10^,^28^. For example, Hopkins *et al*. found that in the sand fiddler crab *Uca pugilator*, MF had a high affinity with RXR, and was synergized with ecdysteriod to stimulate the expression of related genes mediated by the RXR-EcR heterodimer complex^2^. Furthermore, the exogenous MF has been proved to enhance the RXR mRNA level in the green crab *Carcinus maenas* in both *in vitro* and *in vivo* experiments^10^. However, the results of a recent study showed that MF failed to transactivate RXR of the water flea *D. magna*, which was transfected into human hepatocellular carcinoma cells^25^. Thus, the ligands and the regulation mechanism of RXR in crustaceans remain contentious.

In crustaceans, MF has been reported as a naturally occurring terpenoid synthesized by the mandibular organ (MO), whose position has been identified only in the decapod crustaceans^29^. As the immediate precursor of JH, MF participates in various physiological processes of crustaceans^9^,^30^. It has been shown that in the giant freshwater prawn *Macrobrachium rosenbergii*, after larvae being fed *Artemia* enriched with exogenous MF, the adult morphology development in late stage larvae were retarded, showing similar effects to a well-known feature of JH on preventing metamorphosis in insects^28^,^31^. MF is also known as a major crustacean hormone regulating reproductive maturation in crustaceans^29^. For instance, it has been reported that following injection of exogenous MF, the ovarian index of *C. maenas* was significantly enhanced when compared to those control crabs injecting crab saline^10^. Similarly, ovarian maturation of the freshwater crab *Oziotelphusa senex senex* was also reportedly stimulated by the injection of MF^32^. Other functions of MF include stimulating testicular growth^29^, regulating individual morphogenesis and molting^33^,^34^, involving in sex determination^35^,^36^ and participating in osmoregulation^29^. Therefore, coupled with its signaling molecules, MF plays important roles in many physiological processes and is critical to the normal function of crustaceans. However, to date its receptor remains poorly understood, and the molecular basis of the signaling pathway as well as underlying functional mechanisms are largely unknown.

The mud crab *Scylla paramamosain* is a large portunid crab species important to both fisheries and aquaculture along the coasts of southern China as well as in many other Indo-Pacific countries^37^. In a previous study, the nuclear receptor EcR and ecdysone was found to promote ovarian development via regulating vitellogenin (SpVg) expression in the species^38^. However, the functional mechanisms of RXR as another important nuclear receptor and MF on ovarian development of the species have not been clearly studied, especially regarding their signaling pathway. Hence in this study, we cloned three splice variants of RXR from *S. paramamosain* (SpRXR), and quantified the expression and location of SpRXR during ovarian development via quantitative real-time PCR (qRT-PCR) and *in situ* hybridization. Using multiple molecular techniques in combination with both *in vitro* and *in vivo* experiments, the functions of SpRXR and its related signaling pathway were also investigated.

## Results

### SpRXR sequence identification and phylogenetic analysis

**Figure 1:**
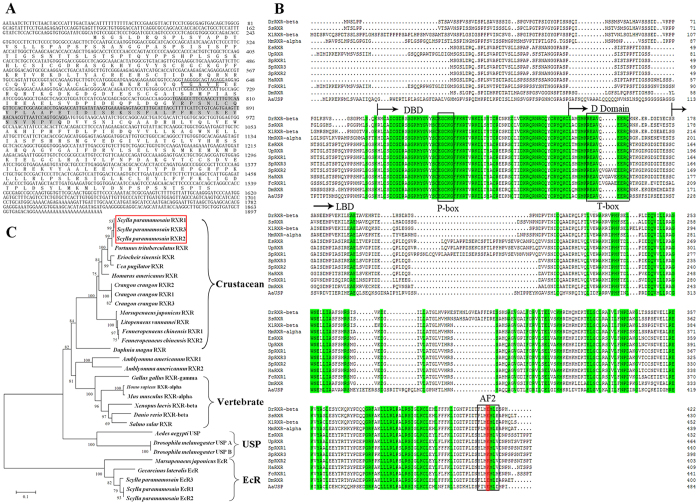
Sequences and phylogenetic analysis of SpRXR. (**A**): nucleotide and deduced amino acid sequences of the SpRXR, a 5 and 42 aa alternative insertion is underlined and shadowed, respectively; (**B**): the alignment of SpRXR amino acid sequences with RXR orthologs from other invertebrate and vertebrate species, the identical aa residues among all the aligned sequences are shaded in green, the P-box, T-box and AF-2 ligand-dependent activation function region are boxed, the different aa residues in the AF-2 region of RXRs or USP between decapod and vertebrate or insect are shaded in red. (**C**): Phylogenetic analysis of RXR, neighbour-Joining tree was produced with the Mega 5.0 software.

Three cDNA isoforms of SpRXR were derived from the alternative deletion sequence, which were designated as SpRXR1, SpRXR2 and SpRXR3. SpRXR1 consisted of a 364 bp 3′-untranslated region (UTR) with a poly A tail and 192 bp 5′-UTR while 5 aa and 42 aa insert sequences were found in the T-box domain of SpRXR2 and the ligand binding domain (LBD) of SpRXR1, respectively (Fig. 1AB). Multiple alignments of the SpRXR isoforms with other RXR/USPs from both vertebrates and invertebrates showed that all the three SpRXR isoforms had a high homology to RXRs of other crustaceans and possessed the typical domain organization of nuclear hormone receptors (Fig. 1B). Interestingly, a conserved acidic amino acid residue (lysine, K) in the ligand dependent activation function 2 region (AF-2) of SpRXR was the same as other decapod RXRs but substituted by glutamic acid (E) in vertebrate RXRs and insect USPs. Moreover, another common feature of crustacean RXRs was that a small insert was always found in the hinge region while a large insert in LBD (Fig. 1B).

To determine the relationships of SpRXR with other homologous sequences, the phylogenetic tree of the RXRs/USPs was constructed using NJ method (Fig. 1C). The unrooted radial NJ tree of RXRs/USPs showed that the RXRs from crustaceans were clustered in a separate clade from those RXRs from vertebrates and USPs from insects. Figure 1C also showed that the crustacean RXRs were closer to the clade of the vertebrate RXRs than that of the insect USPs.

### Expression pattern of SpRXR in different tissues and during ovarian development

**Figure 2:**
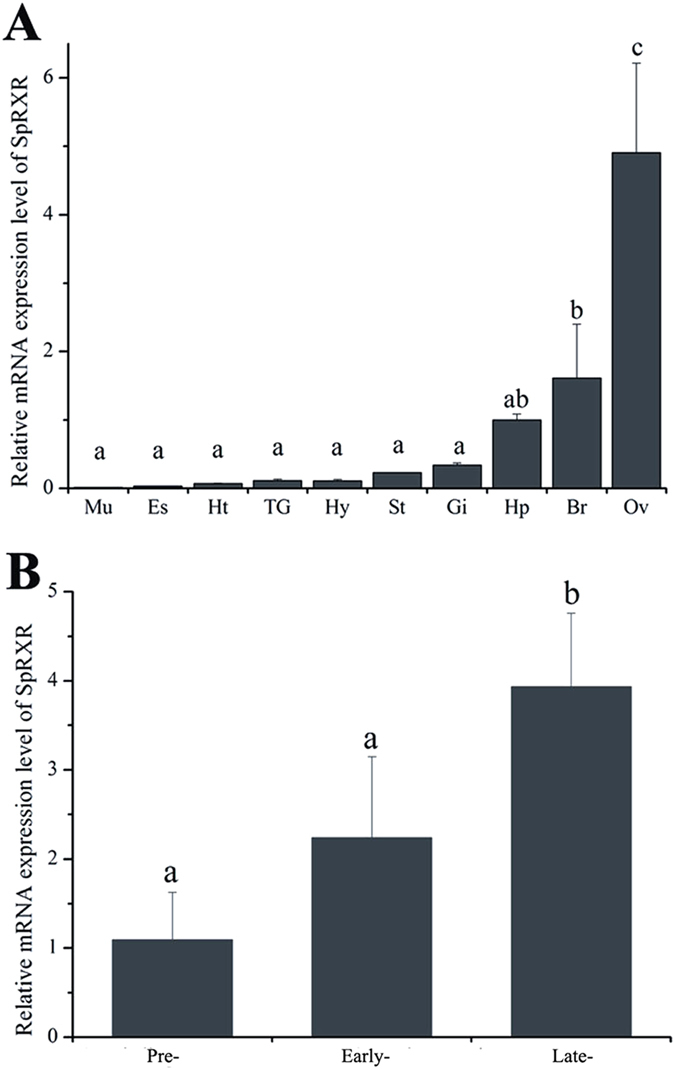
Expression profiles of SpRXR transcripts in various tissues (**A**) and different vitellogenic stages ovary (**B**) in *S. paramamosain*. Expression of the gene encoding *β-actin* was used as a control. The relative abundances of SpRXR transcripts were shown as mean ± SD (N = 3). Mu: muscle, Es: eyestalk, Ht: heart, TG: thoracic ganglion, Hy: hemocyte, St: stomach, Gi: gill, Hp: hepatopancreas, Br: brain, Ov: ovary; Pre-: Previtellogenic stage, Early-: Early vitellogenic stage, Late-: Late vitellogenic stage. Values with different letters on the top of bars are significantly different (P < 0.05).

qRT-PCR analysis demonstrated that the SpRXR (primers used for expression analysis were designed in the conserved region of three isoforms) was extensively expressed in various tissues of the female mud crab, including muscle, eyestalk, heart, thoracic ganglion, hemocyte, stomach, gill, hepatopancreas, brain and ovary (Fig. 2A). However, it was also clear that the expression level of SpRXR gene in the ovary was many times higher than in other tissues (P < 0.05).

To better understand the correlation of SpRXR with ovarian development, the relative abundance of SpRXR transcripts at different ovarian vitellogenic stages were also determined by qRT-PCR. The SpRXR mRNA level was found to show an increasing trend with ovarian development from the pre-vitellogenic to the late vitellogenic stage, with a significant increase detected at the late vitellongenic stage (Fig. 2B).

### Localizing SpRXR in ovary

**Figure 3:**
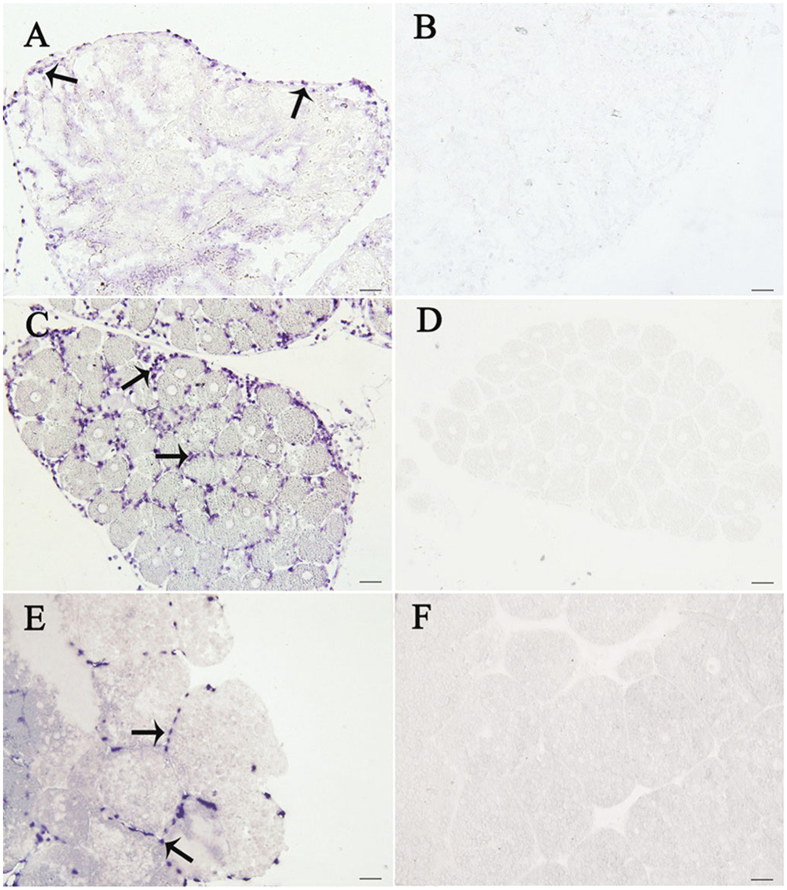
Localization of SpRXR mRNA by *in situ* hybridization in the ovaries of *S*.*paramamosain* at different vitellogenic stages. Arrows indicate the specific SpRXR mRNA signals detected by the antisense riboprobe in ovaries at the previtellogenic stage (**A**), the early vitellogenic stage (**C**) and the late vitellogenic stage (**E**). (**B**,**D**,**F**) show the negative control to (**A**,**C**,**E**), respectively, in which sense riboprobe was used. The scale bar is 50 μm.

*In situ* hybridization of SpRXR mRNA illustrated that in ovaries of the pre-vitellogenic stage, positive signals were localized in the follicular cells distributed along the periphery of the ovarian lobules rather than inside oocytes (Fig. 3A). With the progress of ovarian development, the positive signals were detected in the follicular cells surrounding the oocytes at both the early vitellogenic and the late vitellogenic stage (Fig. 3CE). No positive signal was ever detected in ovaries of the negative controls in which the sense SpRXR riboprobe was used instead (Fig. 3B,D,F).

### Effects of 20E and MF on SpRXR and SpVg expression by *in vitro* experiment

**Figure 4:**
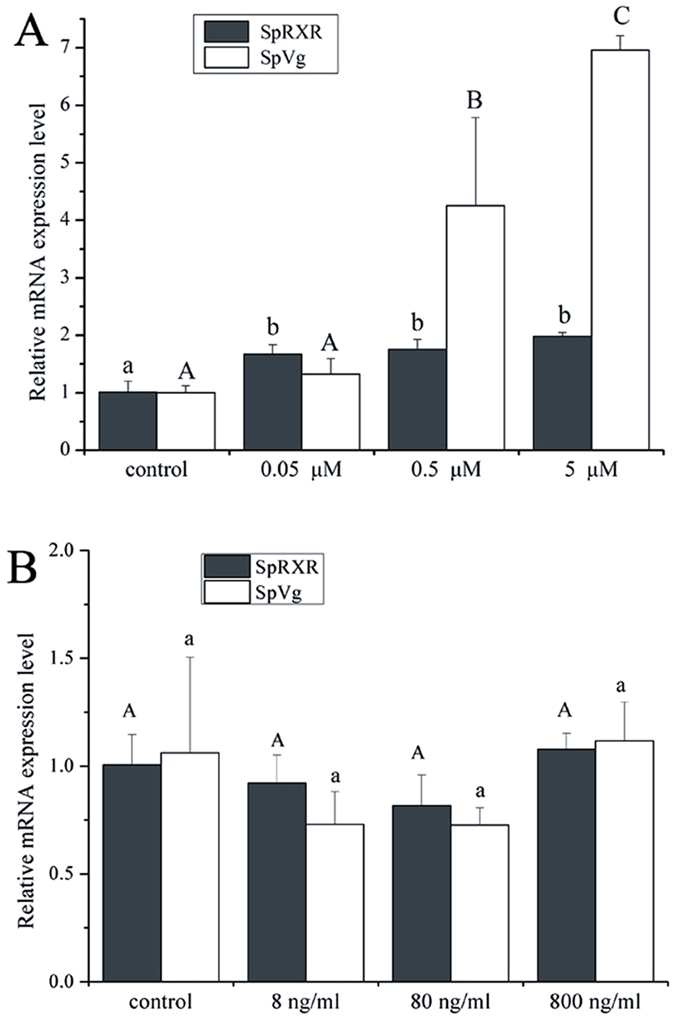
Effects of 20E (**A**) and MF (**B**) on the expressions of SpRXR and SpVg gene in the ovary of *S. paramamosain* by *in vitro* experiment. The explants were sampled at 3 h post-20E addition while 6 h post-MF addition. The relative abundances of genes transcripts were shown as mean ± SD (N = 3). Values with different letters on the top of bars are significantly different (P < 0.05).

Incubation of ovarian explants with 20E significantly induced SpRXR and SpVg: the expression level of SpRXR increased significantly in the ovarian explants treated with 0.05, 0.5 and 5 μM 20E; the transcripts of SpVg were simultaneously significantly increased by four and seven-folds, respectively, when 0.5 and 5 μM 20E were used for incubation (Fig. 4A) (P < 0.05). However, no significant change in the mRNA expression levels of both SpRXR and SpVg were observed in the ovarian explants incubated with exogenous MF, even the MF concentration used was as high as 800 ng/ml (Fig. 4B) (P > 0.05).

### Effects of MF and 20E on genes expression by *in vivo* experiment

**Figure 5:**
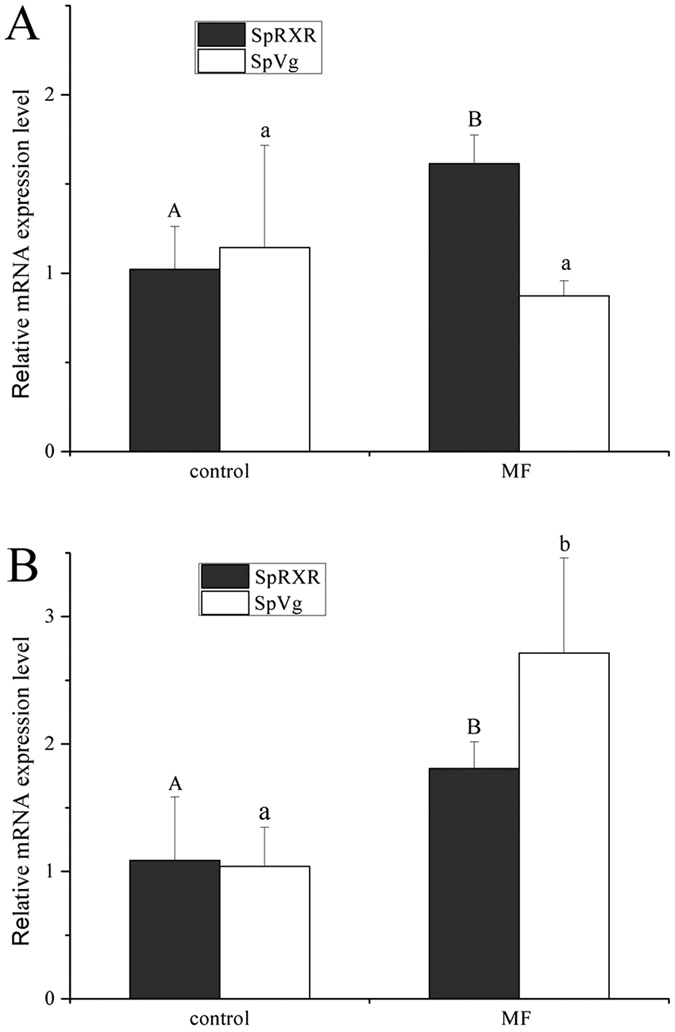
Effects of MF on the expressions of SpRXR and SpVg gene in the ovary of *S. paramamosain* by *in vivo* experiment. The experimental crabs were sampled at 24 h (**A**) and 48 h (**B**) post-injection. The relative abundances of genes transcripts were shown as mean ± SD (N = 3). Values with different letters on the top of bars are significantly different (P < 0.05).

The MF *in vivo* experiment showed that 24 h after MF injection, the expression of SpRXR in the female crabs at the early vitellogenic stage was significantly induced while the SpVg did not show significant difference from that of the control. However at 48 h post injection, both SpRXR and SpVg transcripts increased significantly in response to the MF administration (P < 0.05) (Fig. 5).

**Figure 6:**
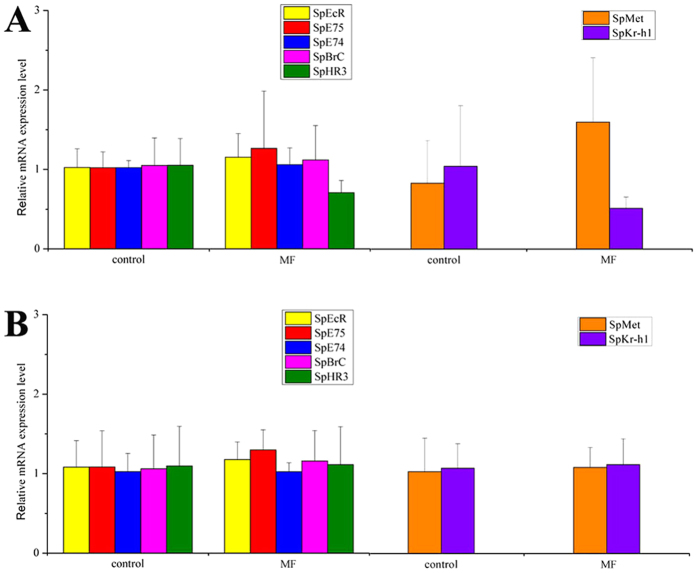
Effects of MF on the expression of ecdysone signaling pathway related genes and the JH response genes in the ovary of *S. paramamosain* by *in vivo* experiment. Experimental crabs were sampled at 24 h (**A**) and 48 h (**B**) post-injection. The relative abundances of genes transcripts were shown as mean ± SD (N = 3).

Differing from the results of SpRXR and SpVg, no significant changes in the expression levels of all identified ecdysone signaling pathway related genes (SpEcR, SpE75, SpE74, SpBr-C and SpHR3) were detected at both 24 h and 48 h in the female crabs receiving MF injection (P > 0.05) (Fig. 6AB). Similarly, of the two JH response genes identified, at 24 h post injection, the expression of SpKr-h1 was lower while SpMet was higher as compared to the control but the differences were both not statistically significant (P > 0.05). At 48 h post-injection, the expression of both SpKr-h1 and SpMet of the treated crabs were almost identical to that of the control crabs (Fig. 6AB).

**Figure 7:**
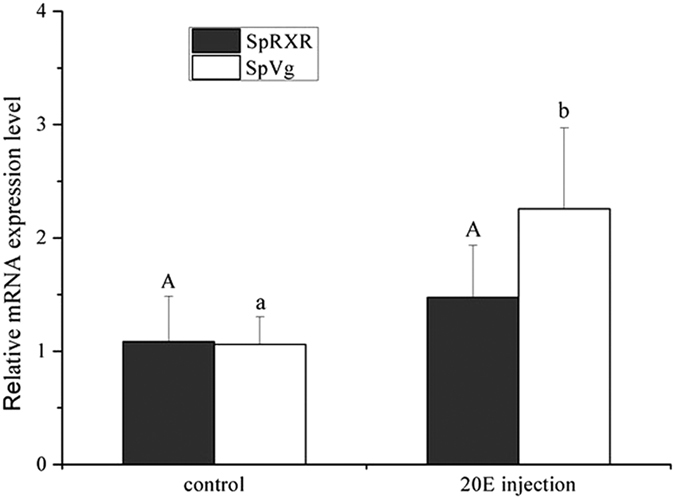
Effects of 20E on the expressions of SpRXR and SpVg gene in the ovary of *S. paramamosain* by *in vivo* experiment. The experimental crabs were sampled at 24 h post-injection. The relative abundances of genes transcripts were shown as mean ± SD (N = 3).

The result of 20E injection showed that at 24 h post injection, 20E significantly stimulated the expression of SpVg. For SpRXR, although the expression was higher in the 20E injected crabs as compared to the control, statistical analysis showed that the difference was not significant (Fig. 7).

## Discussion

As one of the most conserved and widespread steroid/retinoid nuclear receptors, RXR plays important roles in various physiological processes in both invertebrates and vertebrates^4^,^5^,^6^,^7^,^8^,^9^,^10^,^11^,^12^,^13^. While past studies have been mostly focused on the function of RXR in vertebrates and USP in higher holometabolous insects^22^, there are increasing studies in recent years on the involvement of RXR in major physiological processes in crustaceans, such as reproduction, development and limb regeneration^4^,^8^,^10^,^12^,^13^. However, the functional mechanisms of RXR in crustaceans are largely remained unknown.

The present study isolated three isoforms of SpRXR from the mud crab *S. paramamosain*. The deduced amino acid (aa) sequences showed high similarity to RXRs reported from other crustaceans. The three isoforms of SpRXR were produced as a result of an insertion in either the T-box or LBD (Fig. 1B) and no isoform with both insertions was detected, which is different from what was reported in the blue crab *Callinectes sapidus*^4^. The failure to detect a SpRXR isoform with both insertions in the present study might be explained by either its low expression level or in fact no such an isoform exists in *S. paramamosain*. The alternative inserting in the conserved T-box of the isoforms, which is related to mediating interactions of hormone response elements and formation of homodimers or heterodimers, may result in different RXR functions^39^. Similar type of RXR isoforms with a short insertion in the T-box and/or with a long insertion in the LBD have been reported in other crustaceans despite the insertion may vary in size and/or position^4^,^9^,^10^,^39^,^40^,^41^. Unfortunately, different from other nuclear receptors, such as EcR, whose isoforms are studied more comprehensively^42^, up to date the specific functions of different RXR isoforms remain unclear, which warrants further investigation.

In additional to the alternative insertion, multiple sequence alignment demonstrated that the conserved K in the AF-2 region, where is responsible for regulating the ligand-dependent transcriptional activity of the nuclear receptor superfamily^43^, of decapods RXRs is generally substituted by E in vertebrate RXRs and insect USPs (Fig. 1B). It showed that significant divergence existed between the crustacean RXRs and insect USPs/vertebrate RXRs, suggesting that crustacean RXRs might have special transactivation properties that are differed from those of vertebrates and insects^12^,^44^. Phylogenetic analysis indicated that with the exception of the water flea *D. magna*, crustacean RXRs were generally clustered in a separate clade, which was closer to vertebrate RXRs than insect USPs (Fig. 1C). In fact, in the shrimp *Metapenaeus ensis*, it was reported that the LBD of MeRXR was more homologus to the vertebrate RXRs than the insect USPs^12^. Similar results were also reported in the Kuruma shrimp *Marsupenaeus japonicas*^8^ and the green crab *C. meanas*^10^.

Expression profiling of SpRXR in different tissues of *S. paramamosain* revealed that SpRXR was ubiquitously existed in various tissues, however the highest expression level was detected in the ovary, which was many times higher than in all other tissues. It was also found that the SpRXR transcripts were concurrently up regulated in the ovary during ovarian development (Fig. 2B). A similar expression pattern during ovarian development was reported in other crustaceans, including the green crab *C. maenas*^10^, the fiddler crab *U. pugilator*^40^, the American lobster *Homarus americanus*^11^, the swimming crab *Portunus trituberculatus*^15^ and the shrimp *M. ensis*^12^. These results together indicate that RXR probably play important roles in crustacean ovarian development. Similarly in insects, Wang *et al*. also found that the expression level of USP-B in the ovary of mosquito *Aedesa egypti* increased during ovarian maturation^45^.

The control of reproduction in crustaceans is complex and reportedly co-regulated by the ecdysone, crustacean hyperglycemic hormone (CHH) neuropeptides family and MF, which are secreted by Y-organ, X-organ/sinus gland complex and mandibular organ, respectively^11^. In a previous study, we found that coupled with its receptor (SpEcR), 20E, a biologically active form of ecdysone, played important roles in promotion of ovarian maturation in *S. paramamosain*^38^. It was also reported that EcR could bind with RXR to form a heterodimer to activate DNA regulatory elements in the centipede *Lithobius peregrinus*^39^. The injection of RXR dsRNA was also reportedly could knockdown RXR transcript abundance, resulting in significant reduction of ecdysone titers in the German cockroach *Blattella germanica*^46^ and the fiddler crab *U. pugilator*^19^. In this study, the expression profiles of SpRXR in different tissues, during ovarian development, and especially in the *in vitro* experiment in which ovarian explants were incubated with exogenous 20E, showed almost the same pattern as SpEcR^38^. In fact, it has also been reported that without EcR, RXR or USP could not be activated by the ecdysone agonist in cultured cells^47^,^48^; and the binding affinity of ponasterone A, a potent ecdysone agonist, to EcR was remarkably enhanced by the addition of USP^23^. Moreover, *in situ* hybridization in this study located the SpRXR mRNA in the follicular cells of *S. paramamosain*, where SpEcR was also located in a previous study^38^. All these together indicate that SpRXR might be involved in the ecdysone signaling pathway regulating ovarian development in *S. paramamosain*.

However the results of *in vivo* experiment showed that although the expression of SpRXR was higher in the 20E injected crabs than those from the control, no significant difference was detected. In crustaceans, both EcR and RXR are vital in the ecdysone signaling pathway, but their expressional profiles may not be always consistent with the 20E concentration in different development stages^8^. In addition, RXR might also function by interacting with MF, indicates that RXR is not regulated only by 20E^4^. Moreover, it has also been reported that EcR can, at least under certain conditions, regulate the transcription of certain target genes without binding to USP or RXR but formed a homodimer/heterodimer with other nuclear receptors^6^,^7^,^8^,^42^. Therefore, the different results with 20E between the *in vitro* and the *in vivo* experiment may be attributed to the fact that compared to the *in vitro* experiment, the physiological process was much more complex for the *in vivo* experiment in which multiple regulating processes co-existed and interacted, and hence SpRXR might also participate in regulating ovarian development via other ways.

Crustacean ovarian development is characterized by significant vitellogenesis, which involving the synthesis of large quantity of Vg to vitelline as the final product^49^. During the ovarian development, the vitelline was found to gradually accumulate in the ovary of *S. paramamosain*^50^. The increasing trend of SpRXR expression in the ovary of *S. paramamosain* during ovarian development and its localization in the follicular cells where SpVg was also detected by *in situ* hybridization^49^ suggested that SpRXR might take part in regulating transcription or accumulation of SpVg during vitellogenesis. In fact, Tiu *et al*. have reported a correlation between RXR and Vg genes expression profiles in ovary and hepatopancreas, the two Vg synthesis sites, in the American lobster *H. americanus* during the reproductive cycle^11^. More convincingly, several transcription factor binding sites, including USP and RXR, were found in the 5′ promoter region of the Vg gene^51^. Furthermore, two recent studies showed that in the green crab *C. maenas* and the salmon louse *Lepeophtheirus salmonis*, knocking-down the expression of RXR diminished the Vg transcription and yolk granules in the maturing oocytes and diluted the follicular substance, leading to follicular cells filled with vacuoles^6^,^10^.

Although RXR or USP were initially identified as orphan receptors that act as silent binding partners in heterodimeric combinations^8^,^22^, recent studies have speculated that crustacean RXRs are also regulated by MF^2^,^8^,^10^,^16^,^41^. However, in the present study, SpRXR transcription in the ovarian explants was not induced by the exogenous MF, even at a very high concentration (Fig. 4B). A possible explanation for such results could be that SpRXR distributed in the follicular cells actually did not receive MF, MF was alternatively received by receptor(s) in other tissue, organs or cells, which produced secondary factor (s) that induced SpRXR and SpVg expression in the ovary. In fact, it have also been reported that for *in vitro* experiments with the shrimp *M. ensis* and the American lobster *H. americanus*, MF also did not stimulated the expression of response genes as expected^52^,^53^. It was speculated by the authors that their results might be related to high hydrophobicity of MF which has a high affiliation with plastic surfaces used for the *in vitro* incubation^54^,^55^, and then resulted to considerable lower actual concentration than the designated dosages^53^.

Different from the result of *in vitro* experiment, the expressions of SpRXR and SpVg of the female crabs at the early vitellogenic stage were significantly induced by MF injection. A recent study by Nagaraju *et al*. also demonstrated that administration of MF significantly enhanced ovarian index, RXR and Vg transcripts levels in both the ovary and hepatopancreas of *C. maeans*^10^. In crustaceans, MF is secreted by the MO and has been found in the hemolymph of several species as the key endocrine controller for various physiological processes, including molting, reproduction and morphogenesis^29^. As a gonadotropic hormone, MF can stimulates both ovary and testis development and maturation^29^,^30^. During ovarian development of *H. americanus*, a significantly increased MF titer was detected in the hemolymph during secondary vitellogenesis^11^. Injection of MF into the freshwater crab *Oziotelphusa senex* also significantly increased oocyte and follicle diameters and the gonad index in females^32^. In addition, it was reported that implanting MO, where MF is synthesized, from adult male spider crab *Libiniae marginata* to the immature female crabs stimulated ovarian growth^56^. Taken together, these results suggest that MF may serve as the upstream signaling of RXR and is involved in the Vg synthesis and vitellogenesis during ovarian development of crustaceans.

To better understand the functional mechanisms of MF on regulating ovarian development in crustaceans, it is necessary to investigate its signaling pathways. However, so far few research was performed to clarify the comprehensive MF signaling pathway system in decapods. Interestingly, the present study showed that the activation of SpRXR and SpVg was not synchronous following MF injection. In the *in vivo* experiment, SpRXR transcription was significantly induced first at 24 h of MF injection, followed by SpVg at 48 h post MF injection, indicating that the effects of MF inducing the expression of RXR and Vg had a time-lag and between them might involve other hormone response genes. It is known that the RXR can form a complex with EcR and ecdysone to activate the transcription of hormone response genes responsible for vitellogenesis, such as E75, E74, HR3 and Br-C^14^, and that MF can enhance the ecdysone secretion of the *in vitro* Y-organ in crustaceans^57^. On these bases, we once purposed that MF might share the signaling pathway with ecdysone. However, in the present *in vivo* experiment, the ecdysone response genes were not induced by the injection of MF. Eichner *et al*. also reported that in the salmon louse *L. salmonis*, the mRNA levels of ecdysone response genes except EcR were not regulated downward with the RXR knocking down^6^, which supported our new hypothesis that MF might have its own signaling pathway system rather than shared with ecdysone.

In crustaceans, MF is also known as the precursory and active form of insect JH, which plays vital roles in regulation of reproduction in insects and its signaling pathway is better understood^30^,^58^,^59^. For instance, it is known that in insects, Methoprene-tolerant (Met), a basic helix-loop-helix-Per-Arnt-Sim (bHLH-PAS) transcriptional regulator, can form a heterodimer with another bHLH-PAS protein known as steroid receptor coactivator (SRC), in response to JH to activate transcription of the downstream gene, Krüppel-homolog 1 (Kr-h1), to regulate various physiological processes^59^,^60^,^61^. Although less is known about MF signal transduction in crustaceans, Met and SRC homologs have been identified in the water flea *Daphnia pulex* and were found to be responsive to MF, suggesting that signaling pathway system similar to that of JH in insects may exist and conserve in the *Daphnia*^36^. However, in the present study, no significant changes in expression level of SpMet and SpKr-h1was detected in female *S. paramamosain* receiving MF injection. In *D. pulex*, although Met and SRC were sensitive to MF, the expression of Kr-h1 also did not change significantly even under a high titer of MF, suggesting that Kr-h1 is not an MF-responsive gene^36^. It hence indicates that response genes of the MF signaling pathway in decapod crustaceans are likely to be somewhat different from the canonical JH signaling pathway in insects.

**Figure 8:**
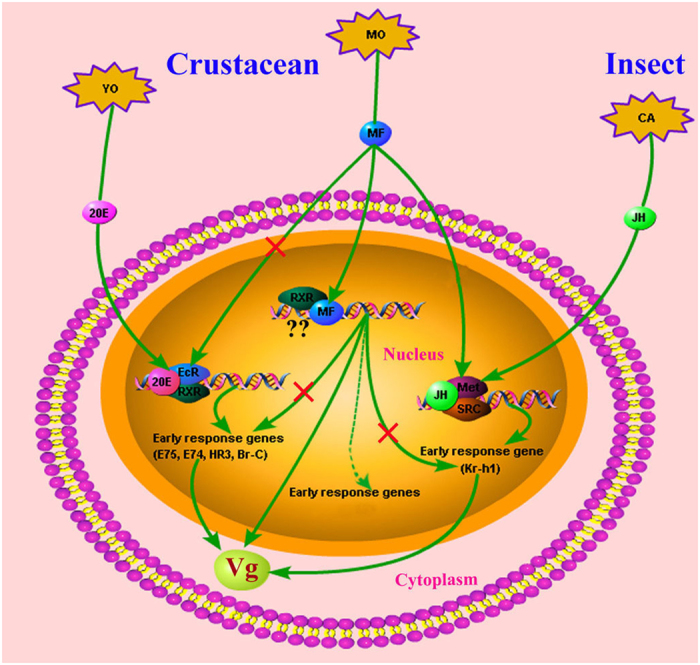
The possible mechanism and signaling pathway of MF and RXR on regulating ovarian development, which was designed based on our study and other literatures. Solid arrows indicate positive for the expressions of responses genes; question mark indicates uncertain factors; dashed arrow means unproved signaling pathway; Solid arrows with × represent no sharing of the related signaling pathway. YO: Y-organ, MO: mandibular organ, CA: corpora allata.

In summary, in the present study, we have isolated three SpRXR variants from the ovary of *S. paramamosain* and the expression levels of SpRXR showed an increasing trend with ovarian development. Similar to SpEcR, SpRXR mRNA was also localized in the follicular cells and significantly higher expression levels of SpRXR and SpVg mRNA in the ovarian explants were induced by exogenous 20E. These results together suggest that SpRXR might be involved in the ecdysone signaling pathway regulating ovarian development in *S. paramamosain*. Meanwhile, it was found that while injection of MF significantly induced the expression of SpRXR and SpVg, no significant effects on the expression of the ecdysone and insect juvenile hormone (JH) signaling pathway genes was detected, indicating MF and RXR might have its own specific signaling pathway rather than sharing with ecdysone or the insect JH in *S. paramamosain*. To our knowledge, the present study presents the first attempt to comprehensively investigate the MF signaling pathway system in decapod crustaceans. Since specific components of MF signaling pathway (Fig. 8) were not identified in this study, further research based on biochemical and molecular assays are recommended for the definition of the functions and molecular basis of MF action in *S. paramamosain*.

## Materials and Methods

### Tissue sampling and RNA isolation

Our study does not involve any endangered or protected species. All animals used in this study have been approved by the Animal Ethical Committee of Xiamen University and experiments were carried out in accordance with the approved guidelines of the university.

Healthy female *S. paramamosain* at different vitellogenic stages (i.e. previtellogenic, early vitellogenic and late vitellogenic stage) were purchased from a local fish market in Xiamen city, China. They were transported to Xiamen University and acclimated for three days in several cement tanks filled with natural seawater (salinity 29 ± 1 ppt; temperature 28 ± 1 °C) before experiments. During the acclimatization, the crabs were fed live clam *Ruditapes philippinarum*, at a ratio of ~30% of their body weight per day.

Past studies have shown that based on vitellogensis, the ovarian maturation in *S. paramamosain* can be divided into three distinct stages^38^,^62^: 1) pre-vitellogenic stage: the ovary appears milky white and contains small oocytes; 2) early vitellogenic stage: the ovary is yellowish to orange in color and oocytes contain yolk granules; and 3) late vitellogenic stage: the ovary appears in orange color and oocytes contain larger yolk granules. Based on above ovarian staging system, the female mud crabs at the early vitellogenic stage were anesthetized on the ice and muscle, eyestalk, heart, thoracic ganglion, hemocyte, stomach, gill, hepatopancreas, brain and ovary tissues were sampled. Additionally, ovarian samples were collected from female crabs with at the pre-vitellogenic and the late vitellogenic stages. All tissue samples were immediately frozen in liquid nitrogen and stored at −80 °C for later total RNA extraction.

For total RNA extraction, the TRIzol reagent was used according to the manufacturer’s instructions (Invitrogen, USA). DNase I was then used to remove residual genomic contaminations. The RNA was subsequently quantified by a ND-2000 NanoDrop UV spectrophotometer (nanoDrop Technologies, Inc., USA) and 2 μg total RNA was reversely transcribed using a reversed first strand cDNA synthesis kit (Fermentas, USA) and stored at −20 °C.

### Cloning, sequencing of SpRXR and identification gene fragments potentially involved in RXR signaling pathway

RXR F and RXR R, the degenerate primers designed for multiple alignment of conserved domain, were used to amplify a partial sequence of SpRXR (Table 1). The full sequence of SpRXR was completed using 3′, 5′ full RACE kit (Takara, Japan) with the specific primers RXR3′ and RXR 5′ (Table 1). PCR products were detected by 1% agarose gel, and the expected DNA fragments were gel-purified and ligated to pMD19-T vectors (Takara, Japan) before being transformed into DH5a competent cells. Clones containing the target fragments were isolated and cultured over night for the subsequent DNA sequencing (Sangon Biotech Co., Ltd, China). Sequence alignment was performed with ClustalW software. The neighbor-joining method (NJ) in MEGA5.0 was used to construct the phylogenetic tree based on protein sequences, and the bootstrapping replication number was 1000.

Based on our transcriptome database for female *S. paramamosain* (data not shown), six cDNA fragments that might be involved in RXR related signaling pathway were identified and re-sequenced. These include JH-inducible genes of SpKr-h1 and methoprene-tolerant (SpMet), as well as ecdysone induced early genes of broad-complex (SpBrC), hormone receptor 3 (SpHR3), SpE75 and SpE74^10^,^29^,^59^,^63^. The full-length cDNA of SpEcR (GenBank accession number JQ821372.1) was cloned in a previous study by us and reported^38^.

### Phylogenetic and sequence analysis of SpRXR

The Sequence alignment was performed with ClustalW software. GenBank accession numbers of the sequences used are: DrRXR-beta, *Danio rerio* (AAI62301.1); XlRXR-beta, *Xenopus laevis* (NP_001081830.1); MmRXR-alpha, *Mus musculus* (NP_001277410.1); SsRXR, *Salmo salar* (ABQ59675.1); EsRXR, *Eriocheir sinensis* (AHF65151.1); UpRXR, *U. pugilator* (AAC32789.3); HaRXR, *H. americanus* (AGI15961.1); FcRXR1, *Fenneropenaeus chinensis* (ACN78601.1), DmRXR, *D. magna* (ABF74729.1); AaUSP, *Aedes aegypti* (EJY57333.1); SpRXR1, *S. paramamosain RXR1* (KT970086.1); SpRXR2, *S. paramamosain RXR2* (JQ821375.2) and SpRXR3, *S. paramamosain* RXR3 (KT970085.1). The neighbor-joining method in MEGA5.0 was used to construct the phylogenetic tree based on protein sequences, and the bootstrapping replication number was 1000. GenBank accession numbers are denoted in front with additional numbers listed as follows: *Portunus trituberculatus* RXR (AGV08303.1); *Crangon crangon* RXR1 (ACO44668.1); *C. crangon* RXR2 (ACO44669.1); *C. crangon* RXR3 (ACO44670.1); *M. japonicas* RXR (BAF75376.1); *Litopenaeus vannamei* RXR (AGS94408.1); *F. chinensis* RXR2 (ACN78602.1); *Gallus gallus* RXR-gamma (NP_990625.1); *Homo sapiens* RXR-alpha (ABB96254.1); *Drosophila melanogaster* USP A (NP_476781.1); *D. melanogaster* USP B (AGB95014.1); *Amblyomma americanum* RXR1 (AAC15588.1); *Amblyomma americanum* RXR2 (AAC15589.1); *S. paramamosain* EcR1 (AFN08659.1); *S. paramamosain* EcR2 (AFN08660.1); *S. paramamosain* EcR3 (AFN08661.1); *M. japonicus* EcR (BAF75375.1) and *Gecarcinus lateralis* EcR (AAT77808.1).

### Expression profiles of SpRXR in different tissues and during ovarian development

To quantify the expression of SpRXR in different tissues and during ovarian development of *S. paramamosain*, qRT-PCR was performed using YRXR F and YRXR R (Table 1), a pair of primers designed based on the sequences of the common domain of different SpRXR isoforms. Two β-actin (GenBank accession numbers: JN975415.1) primers, β-actin F and β-actin R (Table 1), were used to amplify a 183 bp fragment as the internal control^3^. PCR was performed in a 20 μl reaction volume containing 2 μl of cDNA template, 0.8 μl of each primer (10 mM), 10 μl of SYBR premix (Takara, Japan) and 6.4 μl of water. The PCR conditions were as follows: 94 °C for 10 min; 42 cycles of 94 °C for 20 s, 55 °C for 30 s, and 72 °C for 30 s. All samples were analyzed in triplicate.

### *In situ* hybridization

A 325 bp length template of SpRXR was amplified using ovary cDNA by the specific primers of TRXR F and TRXR R (Table 1), which were designed in the common domain of different isoforms. The template was subcloned into the pGEM-T easy vector (Promega, USA) and Digoxigenin-labeled probes were synthesized using a DIG-RNA labeling Kit (Roche, Switzerland). After sampling, the ovarian tissues from the three vitellogenic developmental stages were immediately fixed with 4% paraformaldehyde (PFA) in phosphate-buffered saline (PBS) for one night. The fixed ovarian tissues were then dehydrated through a series of increasing concentrations of ethanol, cleared with xylene and infiltrated with liquid paraffin at 55 °C before finally embedded in paraffin blocks. The blocks were trimmed and sliced to 7 μm on a microtome. *In situ* hybridization was subsequently performed according to the method described in earlier in our previous study^38^. Photographs were taken using an Olympus multifunction microscope (Olympus BX51, Japan).

### *In vitro* experiment with ovarian explants: effects of MF and 20E on gene expression

Ovarian tissues were dissected from the crabs with ovarian development at the early vitellogenic stage and were rinsed nine times with a crab saline solution containing penicillin G at 300 IU/ml and streptomycin at 300 μg/ml (Sigma-Aldrich Chemical Co., USA). The ovarian tissues were then cut into small pieces of ~30 mg and each pieces was placed in a well of 24-well culture plates filled with 0.3 ml of 2 × L15 medium, which contained either 2 μl 20E (Sigma-Aldrich Chemical Co., USA) or MF (Echelon Biosciences, USA) at a designated experimental concentration. Based on previous studies, the dosages used in this study were 0, 0.05, 0.5 and 5 μM for 20E^38^ and 0, 8, 80, 800 ng/ml for MF^10^,^29^,^64^,^65^. Each treatment was triplicated and the culture plates were incubated at 25 °C. Total RNAs from the fragments were extracted 3 h after 20E addition but 6 h after MF addition, which were also based on results reported from previous studies^10^,^38^.

### *In vivo* experiment: effects of MF and 20E injection on gene expression

In order to further investigate the role of MF and 20E on ovarian development and the possible MF related signaling transduction, *in vivo* experiment was conducted to detect the effects of MF and 20E on the expression of SpRXR, SpVg, as well as the ecdysone and JH response genes that might be involved in the MF signaling pathway. For the later, genes fragments related to JH and ecdysone signaling pathway were firstly successfully identified from the transcriptome database of *S. paramamosain*.

For the MF *in vivo* experiment, fourteen female crabs at the early vitellogenic stage of ovarian development (body weight: 235.5 ± 14.3 g) were divided equally into the control and the treatment group. According to previous reports on measurements of MF in hemolymph of crustaceans^29^,^64^,^65^, the crabs assigned to the treatment group received the injection of 20 μl MF at 12 ng/g wet body weight, which translated into about 40 ng/ml in the hemolymph as it has been reported that the total hemolymph volume of a decapod is approximately 30% of its wet weight^26^,^66^. The crabs assigned to the control received the same volume of carrier. All crabs were then maintained as described previously in “Tissue sampling and RNA isolation”. At 24 and 48 h post-injection, three crabs were randomly sampled from each group for the extraction of total RNA from their ovaries.

For the 20E *in vivo* experiment, six female crabs at the early vitellogenic stage were similarly divided equally into two groups of the control and treatment. Based on our previous study^38^, the crabs assigned to the treatment group received injection of 20 μl 20E at 0.2 μg/g wet body weight while the control crabs received the same volume of carrier. All crabs were then maintained as described previously in “Tissue sampling and RNA isolation” and were sampled 24 h after the injection for the extraction of total RNA from their ovaries. The sampling time at 24 h post-injection was based on a previous study showed that exogenous 20E induced the expressions of SpVg in the early vitellogenic crabs 24 h post-injection^38^.

### Statistical analysis

The qRT-PCR data obtained were calculated using 2^−△△Ct^ before subjected to statistical analysis. Student’s t-test and one-way analysis of variance (ANOVA) followed by Duncan’s test were performed to determine any statistically significant differences among treatments, which was set at P < 0.05 level. All statistical analysis was performed using the SPSS 13.0 software (SPSS, Chicago, USA).

## Additional Information

**How to cite this article**: Gong, J. *et al*. The retinoid X receptor from mud crab: new insights into its roles in ovarian development and related signaling pathway. *Sci. Rep*. **6**, 23654; doi: 10.1038/srep23654 (2016).

## Figures and Tables

**Table 1:** Summary of primers used.

Primers	Primer Sequence (5′–3′)	Purpose
RXR F	GGVAARCAYTATGGBGTBTAC	Fragment amplication of RXR
RXR R	GCYTCTCKYTTCATSCCCAT	Fragment amplication of RXR
RXR 3′	GAAGTCTTTTGTTATTGCAAGGGAT	3′ amplication of RXR
RXR 5′	CACTGTCCGCTTGAAGAATCCT	5′ amplication of RXR
YRXR F	CCACACTTCACAGACCTTCCCATA	Quantitative real time PCR for RXR
YRXR R	GGACACCAGCTCAGACAAGACAC	Quantitative real time PCR for RXR
TRXR F	CACCTCCCCAACCCAGTACC	Riboprobe amplication for RXR
TRXR R	CCCGTCTCCTTTGTCACCTTT	Riboprobe amplication for RXR
T7	TAATACGACTCACTATAGGG	Riboprobe amplication for RXR
E75 F	AATGAGGTCCGGGAACGGTTAC	Quantitative real time PCR for E75
E75 R	TGAGGGTGTGGGTGTTGTGAGA	Quantitative real time PCR for E75
E74 F	AGTTCCTGCTGAAGTTGCTG	Quantitative real time PCR for E74
E74 R	CGCTGGTAGTAGTATCTGAGGG	Quantitative real time PCR for E74
HR3 F	CACGGAGACTGTGGAAATGAAA	Quantitative real time PCR for HR3
HR3 R	CACAAGAGCAGAGTACAAGCCTAAC	Quantitative real time PCR forHR3
BrC	GACATGGACGCACTCTTGGA	Quantitative real time PCR for BrC
BrC	GGGAGGAGCTGTTTCTTGGT	Quantitative real time PCR for BrC
EcR F	AAGAACAAAAGACTCCCACCATT	Quantitative real time PCR for EcR
EcR R	TCTCTCACTTACAGCCGACAGG	Quantitative real time PCR for EcR
Met F	GCCTCGACAAGACCTCCACT	Quantitative real time PCR for Met
Met R	CCCGACAGCCTCTGCTAAAT	Quantitative real time PCR for Met
Kr-h F	TCAGCGTCAAGGAGAACCTCA	Quantitative real time PCR for Kr-h
Kr-h R	GGCCAGACTGCACGAATGTC	Quantitative real time PCR for Kr-h
β-actin F	GAGCGAGAAATCGTTCGTGAC	Internal control
β-actin R	GGAAGGAAGGCTGGAAGAGAG	Internal control
